# Comparison of SPEED, S-Trap, and In-Solution-Based Sample Preparation Methods for Mass Spectrometry in Kidney Tissue and Plasma

**DOI:** 10.3390/ijms24076290

**Published:** 2023-03-27

**Authors:** Evelyn M. Templeton, Anna P. Pilbrow, Torsten Kleffmann, John W. Pickering, Miriam T. Rademaker, Nicola J. A. Scott, Leigh J. Ellmers, Christopher J. Charles, Zoltan H. Endre, A. Mark Richards, Vicky A. Cameron, Moritz Lassé

**Affiliations:** 1 Christchurch Heart Institute, Department of Medicine, University of Otago, Christchurch 8014, New Zealand; 2 Research Infrastructure Centre, Division of Health Sciences, University of Otago, Dunedin 9016, New Zealand; 3 Department of Nephrology, Prince of Wales Hospital, Sydney, NSW 2031, Australia; 4 Cardiovascular Research Institute, Department of Cardiology, National University of Singapore, Singapore 119077, Singapore

**Keywords:** mass spectrometry, kidney, plasma, sample preparation techniques, proteomics, renal, suspension trap, SPEED, quantitative proteomics, SWATH-MS

## Abstract

Mass spectrometry is a powerful technique for investigating renal pathologies and identifying biomarkers, and efficient protein extraction from kidney tissue is essential for bottom-up proteomic analyses. Detergent-based strategies aid cell lysis and protein solubilization but are poorly compatible with downstream protein digestion and liquid chromatography-coupled mass spectrometry, requiring additional purification and buffer-exchange steps. This study compares two well-established detergent-based methods for protein extraction (in-solution sodium deoxycholate (SDC); suspension trapping (S-Trap)) with the recently developed sample preparation by easy extraction and digestion (SPEED) method, which uses strong acid for denaturation. We compared the quantitative performance of each method using label-free mass spectrometry in both sheep kidney cortical tissue and plasma. In kidney tissue, SPEED quantified the most unique proteins (SPEED 1250; S-Trap 1202; SDC 1197). In plasma, S-Trap produced the most unique protein quantifications (S-Trap 150; SDC 148; SPEED 137). Protein quantifications were reproducible across biological replicates in both tissue (R^2^ = 0.85–0.90) and plasma (SPEED R^2^ = 0.84; SDC R^2^ = 0.76, S-Trap R^2^ = 0.65). Our data suggest SPEED as the optimal method for proteomic preparation in kidney tissue and S-Trap or SPEED as the optimal method for plasma, depending on whether a higher number of protein quantifications or greater reproducibility is desired.

## 1. Introduction

Mass spectrometry (MS) is a powerful tool for investigating complex biological systems [1] and discovering novel peptide biomarkers [2], capable of identifying and quantifying thousands of proteins in a wide range of biological samples [3]. MS helps us to better understand kidney disease [4], but researchers require a reliable and efficient method to prepare kidney tissue for MS analysis.

Most MS proteomic studies employ a bottom-up strategy in which complex protein mixtures or purified proteins are enzymatically proteolyzed into peptides for MS analysis. Peptide ion intensities, charge states, and chromatographic peak profiles are used to infer the identity of peptide sequences, and from these identified peptides, proteins are then identified and quantified [5]. The depth of proteome coverage achieved by MS is directly correlated to the sample preparation technique; therefore, efficient protein extraction and proteolysis from kidney tissue are critical for adequate protein identification and quantification [6,7].

A key step in sample preparation is the complete lysis of tissues and cells, and solubilization/preservation of proteins to reflect the in vivo state of the proteome [8]. In-solution digestions are based on the extraction and solubilization of proteins using digestion-compatible detergents or high concentrations of urea, followed by dilution to a range of concentrations that does not inhibit tryptic digestion. The use of urea is limited due to its ineffective extraction of proteins from hard-to-lyse samples [9], and as an alternative, sodium dodecyl sulfate (SDS) or other detergents such as sodium deoxycholate (SDC) are commonly employed due to their superior lysis efficacy. However, high concentrations of detergent interfere with downstream sample processing steps such as enzymatic protein hydrolysis and liquid-chromatography (LC) coupled with MS [10]. Therefore, detergent-based preparation methods necessitate the removal of interfering detergents before MS by bead-based purification [11], filtration [10], suspension-trapping (S-Trap) [12], or precipitation [13]. Studies have compared the strengths and weaknesses of each of these strategies [7,14,15,16], concluding that there is no universal sample preparation method that performs optimally for all types of tissues or biofluids [14].

Since its development in 2014, S-Trap has gained popularity due to its ability to process SDS-containing protein lysates in a much shorter time than traditional in-solution methods [7]. In S-Trap, 5% SDS is used to lyse proteins, and a fine protein particulate suspension is formed through the addition of buffer solution and phosphoric acid [12]. Samples are loaded onto a quartz spin column containing submicron pores, which capture proteins. Residual SDS must be washed away before captured proteins can be digested with the protease of choice on the column, resulting in free peptides that can be eluted [12] and subjected to LC–MS analysis. This technology confers a highly efficient method of detergent removal when compared to filter-aided sample preparation, dramatically decreasing the time required for centrifugation of samples and reducing the number of sample handling steps [7,16].

Recently, Doellinger et al. described sample preparation by easy extraction and digestion (SPEED), a novel method that circumvents the necessity for detergent removal before LC–MS analysis [9]. SPEED employs trifluoroacetic acid for protein extraction rather than detergents or chaotropic agents such as urea and consists of three simple steps: (i) acidification, (ii) neutralization, and (iii) digestion. As additional sample processing steps are associated with diminished reproducibility, increased risk of contamination biases, and attendant losses [17,18], SPEED may reduce experimental variation and provide an efficient, reproducible method for sample preparation, especially valuable for medium- to large-scale clinical applications.

While the efficacy of SPEED has been validated in lung and liver tissue samples [9], its performance in kidney tissue has not yet been assessed. Therefore, it is important to trial this preparation method in kidney tissue to determine whether it is an efficient strategy for producing high-quality MS data. Furthermore, SPEED has not been validated in plasma, a biofluid of interest for researchers aiming to discover circulating biomarkers of kidney injury [19]. Unlike kidney tissue, proteins are already in solution in plasma [20], meaning that the choice of agent for lysis and protein extraction (detergent, acid) may have less of an impact on the overall performance.

This study provides a direct quantitative comparison of the recently developed SPEED method with two well-established detergent-based methods in both plasma and kidney tissue to determine the most effective proteomic preparation method for each sample type. We compared three methods of sample preparation using ovine kidney cortical tissue and EDTA plasma: a traditional in-solution preparation method utilizing SDC, S-Trap, and the novel SPEED method. The quantitative and qualitative performance of each method was compared using label-free quantification (sequential window acquisition of all theoretical mass spectra, SWATH-MS).

## 2. Results and Discussion

This study compared the quantitative performance of traditional in-solution digestion using SDC (SDC), suspension trapping (S-trap), and a novel detergent-free approach (SPEED) using SWATH-MS and a standardized set of sheep kidney tissue and plasma samples.

### 2.1. Protein and Peptide Quantification Using SWATH

**Figure 1:**
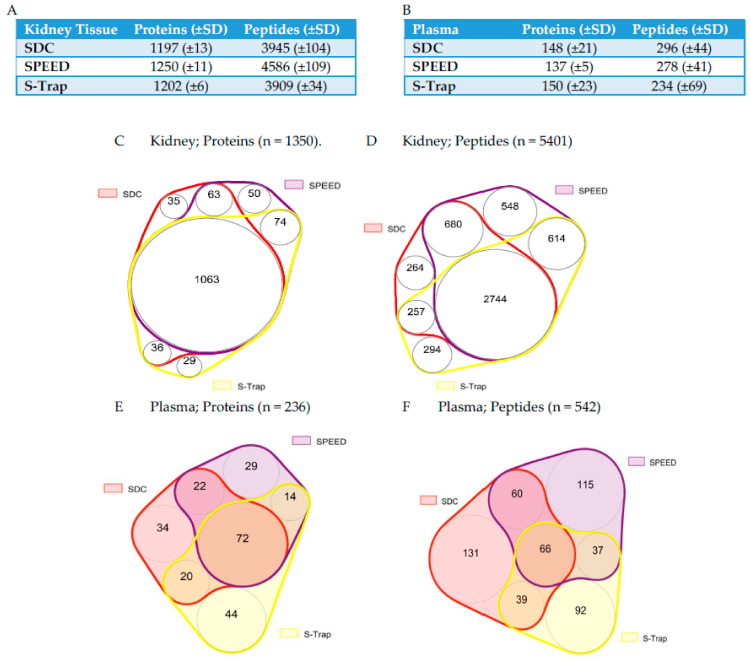
Summary tables (**A**) and proportional Venn diagrams (**B**) indicating the number of (**C**) proteins and (**D**) proteins quantified in kidney tissue, and number of (**E**) proteins and (**F**) peptides quantified in plasma by each preparation method using SWATH-MS. Areas of overlap indicate proteins/peptides quantified in SWATH by more than one preparation method.

In kidney tissue, S-Trap quantified proteins across five orders of magnitude of mass spectrometry signal, whilst SDC and SPEED quantified proteins across four orders (Supplementary Figure S1). SPEED sample preparation resulted in the greatest number of protein (*p* = 0.012) and peptide (*p* = 0.002) quantifications for sheep kidney tissue samples in SWATH-MS (1250 proteins, 4586 peptides), followed by S-Trap (1202 proteins, 3909 peptides) and SDC (1197 proteins, 3945 peptides; Figure 1A,C,D). Whilst this difference in the number of quantified proteins is statistically validated, it should be noted that this difference translates to <5% when compared between the two methods, which quantified the highest (SPEED; 1250) and lowest (SDC; 1197) number of proteins. There was substantial overlap in protein and peptide quantifications across methods for kidney tissue samples, with 91.6% of proteins and 79.5% of peptides being quantified in at least two methods. In contrast, in plasma, there was only a modest overlap of quantifications between methods, with approximately half of proteins and peptides quantified in at least two methods (54.2% and 37.5%, respectively). In plasma, all methods quantified a similar number of proteins (*p* = 0.870) and peptides (*p* = 0.382) across five orders of magnitude of MS signal (Supplementary Figure S1). Nominally, S-Trap yielded the greatest number of robustly quantified proteins (150 proteins, 234 peptides), SDC an intermediate number (148 proteins, 296 peptides), and SPEED the fewest (137 proteins; 278 peptides; Figure 1B,E,F).

The superior performance of SPEED for protein extraction from kidney tissue may be attributed to the cell lysis step. In general, protein extraction from tissue requires lysis of connective tissue and membranes, which is not required for plasma analysis [9]. Detergent-based methods such as S-Trap and SDC require additional mechanical disruption to facilitate adequate lysis, such as grinding, heat [21], or ultra-sonication [9,22]. In comparison, SPEED achieves tissue lysis through exposure to pure trifluoroacetic acid. Trifluoroacetic acid is an excellent solvent for proteins [23], achieving tissue and membrane disruption and protein extraction by complete sample dissolution, even in lysis-resistant samples [9]. Thus, the complete dissolution of kidney tissue achieved by trifluoroacetic acid in this experiment [9] may have contributed to the greater number of peptides and proteins quantified by SWATH-MS in SPEED than in the detergent-based methods. On the other hand, lysis is not required in plasma, so the detergent-based methods (SDC and S-Trap) may have solubilized proteins to a similar extent as the trifluoroacetic acid in SPEED.

### 2.2. Comparing Quantifications across Methods

**Figure 2:**
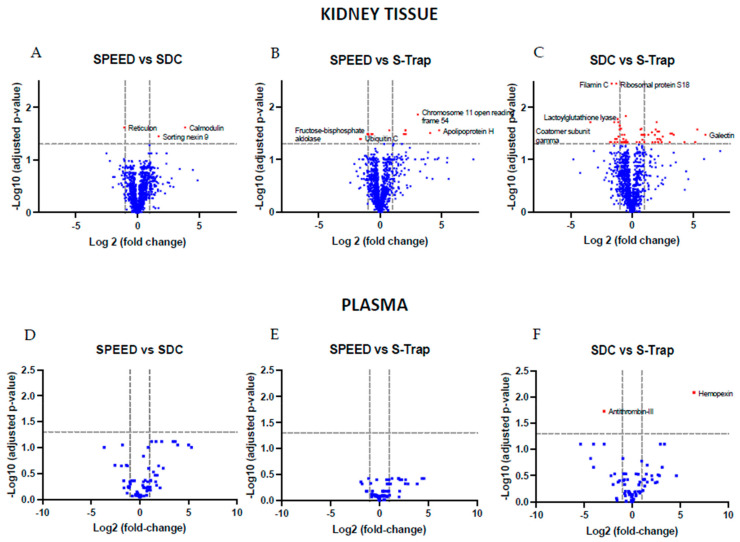
Volcano plots displaying false discovery rate adjusted *p*-values and fold-changes in SWATH protein intensity between methods in kidney tissue (**A**–**C**) and plasma (**D**–**F**). Proteins displayed in red represent those meeting the threshold for differential quantification (a fold-change with an adjusted *p*-value ≤ 0.05); the horizontal grey dashed line represents *p*(adjusted) = 0.05. The vertical grey dashed line represents a fold-change of two. In (**A**,**B**,**D**,**E**), protein fold-changes for SDC and S-Trap, respectively, are in reference to SPEED. In (**C**,**F**), protein fold-changes in S-Trap are with reference to SDC.

We compared quantified protein intensities from each method to the protein intensities in each of the other two methods to test for the differential performance of the methods (adjusted *p* ≤ 0.05, Figure 2). While mostly consistent between sample preparation methods, each method also yielded several significantly different protein abundance patterns. The two methods with the greatest number of differentially quantified proteins were SDC and S-Trap. In kidney tissue, SDC quantified 34 proteins (3.2% of all quantified proteins) at a greater abundance than S-Trap, and 33 (3.1%) proteins at a lower abundance. In plasma, SDC quantified one protein (1.4%) at a greater abundance, and one protein (1.4%) at a lower abundance than S-Trap. A small degree of differential quantification of sets of proteins across methods is not unexpected due to differences in protein extraction and digestion, which may bias each method to a distinct portion of the proteome.

**Figure 3:**
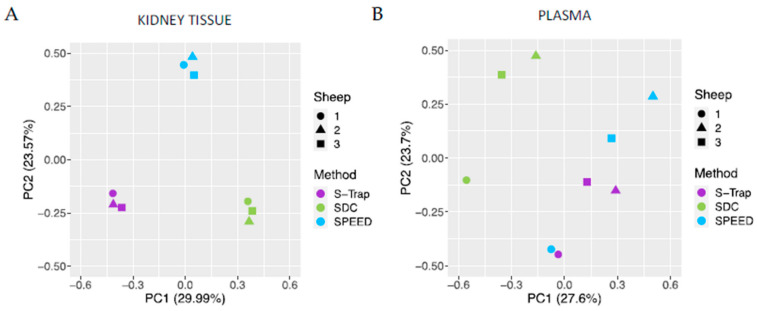
Principal component analysis of SWATH protein intensity data in (**A**) kidney tissue and (**B**) plasma. The color of data points indicates the sample preparation method used, and the shape of data points indicates the identity of the sheep.

Principal component analysis (PCA) of the protein intensity data in kidney tissue produced three distinct clusters revealing that the sample preparation method, rather than the biological specimen (individual sheep), was the leading cause of variation between the samples (Figure 3A). In plasma, the PCA of the protein intensity data produced less distinct clusters of samples, indicating greater variability in protein intensity that may be influenced by other factors, including the complexity of the composition of plasma and biological variability between the individuals, in addition to the sample preparation method (Figure 3B). However, there is distinct segregation in Component 1 between the SDC method (shown in green) from the other two methods, indicating that the variability in this component may be due to the performance of the sample preparation method.

The Core and Comparative gene set enrichment analysis workflow in Ingenuity Pathway Analysis software (Qiagen Inc., https://www.qiagenbioinformatics.com/products/ingenuity-pathway-analysis (accessed on 20 July 2022)) was used to perform functional analysis on the proteins quantified from each preparation method. Overall, there was a striking consistency in the functional pathways identified from the proteins yielded by each method. In both kidney tissue and plasma, the top enriched canonical pathways were consistent across all methods, with no unique pathways identified for any of the preparation methods, setting a conservative Benjamini–Hochberg corrected *p*-value of < 0.001 [24,25]. In kidney tissue, the top three enriched canonical pathways across all methods were Mitochondrial Dysfunction, Oxidative Phosphorylation, and the Sirtuin Signaling Pathway (*p*-values < 0.001). In plasma, the top three enriched canonical pathways across all methods included the Acute Phase Response, Liver X Receptor/Retinoid X Receptor (RXR) Activation, and Farnesoid X Receptor/RXR Activation (*p*-values < 0.001).

### 2.3. Reproducibility of SWATH Quantifications

To compare the reproducibility of the sample preparation methods, we compared the overlap in proteins quantified between the two healthy sheep (biological replicates) in kidney and plasma samples separately (Supplementary Figures S2 and S3). In kidney tissue, between 88 and 91% of proteins (1233–1292) were quantified in both biological replicates. SPEED had 91% overlap, while SDC had 88%. In plasma, the overlap of proteins was much lower (potentially indicating greater biological variation in plasma compared with kidney tissue), with only 42–44% of proteins (188–205) quantified in both biological replicates. Both SDC and S-Trap methods had the same overlap of proteins quantified (44%), while SPEED had marginally less overlap (42%).

We compared the quantitative reproducibility of each method between biological replicates and found a strong correlation (Pearson R^2^ > 0.85) between protein intensities generated by each method in kidney tissue (Supplementary Figure S2). For plasma, S-Trap displayed the weakest correlation of protein intensities, with an R-squared of 0.64 compared to SDC (0.76) and SPEED (0.84; Supplementary Figure S3). Overall, these results highlight that there is considerably less biological variability in the measurement of the kidney proteome versus the plasma proteome. Plasma is a highly complex and dynamic biofluid and is, therefore, a challenging matrix for proteome profiling. Plasma has an extremely wide dynamic range of protein abundances, with > 99% of the total protein mass content in plasma being composed of a set of highly abundant proteins [26], meaning that tryptic peptides from abundant proteins dominate MS analysis and mask less abundant peptides [20,26]. These factors may have contributed to the relatively low percentage of quantified protein identities common to both sheep in plasma. However, among the proteins in common, the protein intensities were highly consistent across the methods.

### 2.4. Efficiency of Proteolysis

**Figure 4:**
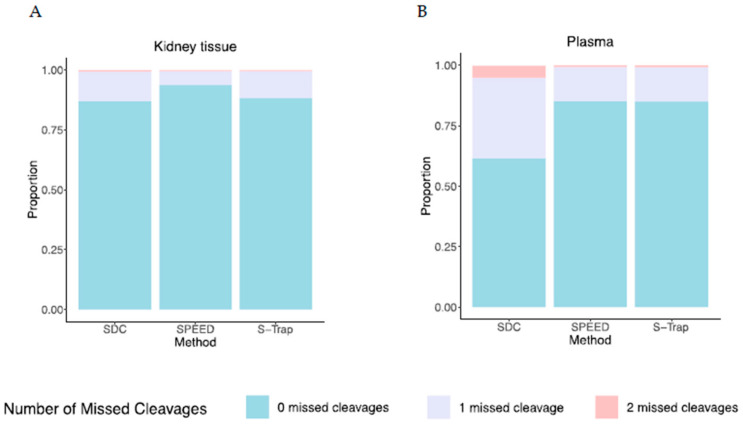
Trypsinization efficiency across preparation methods. The proportion of total detected peptides with missed cleavages for samples prepared using the SDC, SPEED, and S-Trap methods in (**A**) kidney cortical tissue and (**B**) plasma. Proportions displayed are the mean across three biological samples.

We assessed the efficiency of tryptic proteolysis for each method by recording the percentage of peptides identified in Data Dependent Acquisition MS with missed cleavage sites (Figure 4). In kidney tissue, SPEED had the lowest percentage of missed cleavages (6.2% of peptides had at least one missed cleavage site), followed by S-Trap (11.5%) and SDC (12.7%). For plasma, SPEED and S-Trap had similar percentages of missed cleavages (14.6% and 15.2%, respectively), while for SDC, 37.8% of peptides had at least one missed cleavage site, indicating markedly less efficient proteolysis of proteins. SWATH-MS quantifications showed no evidence for greater variability in the quantification of proteins that contained peptides with missed cleavages when compared to the same proteins quantified with only fully tryptic peptides (Supplementary Figure S4). This could be because many peptides (most of which are tryptic) contribute to the quantification of each protein. Therefore, by quantifying many peptides from a single protein, the accuracy and reproducibility of the protein quantification are preserved, even if a proportion of peptides contain missed cleavages.

The percentage of the protein amino acid sequence covered by peptides was compared between methods, including for proteins uniquely detected by each method (Supplementary Figure S5). In kidney tissue, SPEED had the greatest protein coverage (median 14.5%, IQR 8.2–23.2%; *p* < 0.001), followed by SDC (median 13.9%, IQR 7.9–22.6%) and S-Trap (13.2%, IQR 7.6–21.3%). In plasma, SPEED resulted in the highest median protein coverage (median 11.3%, IQR 6.3–20.5%; *p* < 0.001), followed by SDC (11.0%, IQR 6.0–19.3%) and S-Trap (10.2%, IQR 6.0–18.1%).

### 2.5. Physical Characteristics

An important consideration for MS experiments that are interested in a specific protein or set of proteins—for example, membrane proteins or basic/acidic proteins—is whether certain preparation methods are biased towards particular physical characteristics. We compared the effect of the lysis buffer (SDC, SDS for S-Trap, and trifluoroacetic acid in SPEED) on the protein hydrophobicity profile between methods. All preparation methods displayed a bias toward hydrophilic proteins in both tissue and plasma, possessing a mean grand average of hydropathy (GRAVY) score less than zero (Supplementary Figure S6E, Table S6). The mean GRAVY score did not differ between methods in tissue or plasma, indicating a similar extraction efficiency for hydrophobic and hydrophilic proteins by each method (tissue *p* = 0.83; plasma *p* = 0.99).

All methods displayed a similar distribution of peptide identifications in both kidney tissue and plasma based on their net charge and isoelectric points (p*I;* Supplementary Figure S6, Supplementary Tables S1–S6). In tissue, most peptides had a p*I* between 4.2 and 7.3, while in plasma, most peptides identified had a p*I* between 3.9 and 6.4. Additionally, in both plasma and tissue, SDC samples displayed a modest shift towards longer peptides and those with greater molecular weight in their cumulative distribution. This difference is likely a consequence of the decreased proteolysis efficiency observed in samples prepared using the SDC method (most strikingly in plasma).

### 2.6. Practical Considerations

Time is a significant consideration when performing large-scale bottom-up MS experiments [9]. Of the three trialed methods, SPEED required the least hands-on time for preparing samples for SWATH-MS in both kidney tissue and plasma, while SDC was the least time-efficient. SPEED also utilized the fewest number of processing steps, as it avoids the need for tissue homogenization and detergent removal. However, when considering total throughput time, including incubations and digestion, S-Trap is the quickest method. The most time-consuming hands-on step across all three methods was the desalting of peptides, which could be reduced using automation through the use of a trap column or filter prior to the analytical column in a liquid chromatography setting or the use of a commercially available purification device [27]. It is important to note that this desalting step is optional in S-Trap and could be omitted to further save time. Additionally, omitting the desalting step in S-Trap may reduce potential sample loss introduced by this process—a limitation that must be noted in this study.

### 2.7. Limitations

This study has several limitations. First, because we prioritized performing biological replicates and MS injection triplicates, we were unable to evaluate the reproducibility of the peptide extraction in each sample using technical replicates. Second, only certain aspects of the sample preparation protocols were standardized. This was a deliberate choice to enable a comparison of the sample preparation workflows in their entirety, as described in the literature [9,12,28]. This better simulates the implementation of the sample preparation workflows, which many laboratories would use. For example, we did not standardize the temperature and incubation time for trypsin digestion, which could have impacted the efficiency of tryptic proteolysis across the methods. Instead, we used the incubation parameters recommended for each method in the literature to allow us to test the entire methodology workflow.

## 3. Conclusions

Efficient and reproducible sample preparation is critical for accurate protein quantification [6] in bottom-up proteomics. This study is the first to provide a direct quantitative comparison of the recently developed SPEED method with two well-established detergent-based methods to determine the most efficient and effective proteomic preparation method for both plasma and kidney tissue. The effective performance of SPEED and S-Trap indicates that these methods would generate the most complete and accurate data when utilized in proteomic research pertaining to kidney tissue or plasma.

Each sample preparation method possesses distinct advantages and disadvantages that must be considered in the context of specific experiments [7,14,15,16]. Of the trialed sample preparation methods, SPEED was the most time-effective and yielded the greatest number of reproducible protein quantifications in kidney tissue. In plasma, S-Trap yielded the greatest number of reproducible protein quantifications, but demonstrated the lowest degree of reproducibility in protein quantifications between biological replicates. SPEED did not quantify as many proteins as S-Trap in plasma but exhibited the greatest reproducibility for protein quantifications. This sample type-dependent performance highlights the need for experiment-specific optimization based on the biomaterial being analyzed [14]. Therefore, SPEED provides the best balance of performance and time for processing kidney tissue samples compared to the other bottom-up preparation methods trialed in this study. Our data indicate S-Trap or SPEED as the optimal method for plasma, depending on whether a higher number of protein quantifications or greater reproducibility is desired.

## 4. Materials and Methods

### 4.1. Animals

Paired kidney cortex and plasma samples were collected from two healthy sheep and one sheep with pacing-induced acute decompensated heart failure (ADHF), as previously described [24]. The study protocol was approved by the Animal Ethics Committee of the University of Otago–Christchurch (#C23/10 and AUP 19–77). Immediately prior to protein preparation, small pieces of frozen kidney cortex were ground using a mortar and pestle and chilled with liquid nitrogen. Frozen plasma was thawed on ice and centrifuged for 5 min at 13,000× *g* at 10 °C to remove any particulate matter.

### 4.2. Sample Preparation

SPEED [9], S-Trap [12], and SDC methods were performed on kidney (n = 3) and plasma (n = 3) samples, resulting in a total of 18 protein preparations for MS. To minimize variability between the sample preparation methods, we consistently used 150 µg protein material as input for trypsinization, corresponding to approximately 2.5 µL of plasma [29]. Bicinchoninic acid (BCA) assay was used to quantify protein content in S-Trap and SDC samples, while turbidity was used to measure protein content in SPEED samples due to incompatibility with the chemistry of BCA assays. Turbidity could not be used for S-Trap and SDC as proteins remain in solution, whereas proteins precipitate and form a turbid solution in SPEED. We employed the same method of reduction and alkylation using Tris (2-carboxyethyl)phosphine (TCEP) and 2-Chloroacetamide (CAA) and used a fixed ratio of protein:trypsin (25:1) across protocols. The same tissue homogenization protocol was performed for the S-Trap and SDC methods using their respective lysis buffers, while SPEED did not require tissue homogenization (however, it required kidney tissue to be ground in liquid nitrogen).

#### 4.2.1. SPEED

The SPEED protocol was performed as described by Doellinger et al. [9]. Ground, frozen kidney (100 mg) or plasma (2.5 µL) was added to trifluoroacetic acid (TFA; Sigma-Aldrich, St. Louis, MO, USA) at a 1:4 *v/v* sample:TFA ratio and incubated at room temperature with occasional mixing by inversion. Kidney was incubated for 20 min until all tissue was dissolved, and plasma was incubated for 10 min. To neutralize the pH, 2 M Tris(hydroxymethyl)aminomethane (TRIS) Base (Sigma-Aldrich) was added at 10× the volume of TFA. Samples were reduced using Tris (2-carboxyethyl)phosphine (TCEP; Sigma-Aldrich) at a final concentration of 10 mM and alkylated using 2-Chloroacetamide (CAA; Sigma-Aldrich) at a final concentration of 40 mM by incubation at 95 °C for 5 min. For kidney samples, the protein concentration was determined by turbidity measurement at 360 nm compared against MacFarland turbidity standards (bioMérieux, OT-70900, Marcy-l’Etoile, France) [9] as described in the Supplementary Materials. For trypsinization, 150 µg of protein was used and adjusted to 0.75 µg/µL in a 10:1 *v/v* mix of 2 M Tris Base and TFA, then diluted 1:5 using high-performance liquid chromatography (HPLC) grade water (Fisher Chemical, Waltham, MA, USA). Digestion was carried out for 20 h at 37 °C at 600 rpm on a Thermomixer, using a protein:trypsin ratio of 25:1 (Sequencing Grade Modified Trypsin, PRV5111, Promega, Fitchburg, WI, USA). To quench trypsinization, TFA was added to a final concentration of 2%.

#### 4.2.2. S-Trap

Ground, frozen kidney samples were homogenized, and kidney and plasma samples were sonicated in S-Trap lysis buffer, as described in Supplementary Materials. Samples were clarified by centrifugation at 15,000 rpm for 10 min. Homogenized kidney samples (150 μg protein) were made up to a volume of 50 μL to achieve 1× S-Trap lysis buffer and simultaneously reduced and alkylated using TCEP and CAA as described above. The ‘S-Trap midi protocol long 4.1′ (Protifi, Farmingdale, NY, USA) was performed according to manufacturer’s instructions with minor adjustments. Briefly, phosphoric acid (Fisher Scientific, Hampton, NH, USA) was added to the SDS lysate to a final concentration of 1.2% before S-Trap binding buffer (350 μL, 90% aqueous methanol (Fisher Scientific)) containing 100 mM Triethylammonium bicarbonate (TEAB; Sigma-Aldrich) was added to the acidified lysate and loaded onto an S-Trap column. Bound proteins were washed, and S-Trap digestion buffer (50 mM TEAB) containing trypsin at a protein:enzyme ratio of 25:1 was added directly to the column. Samples were incubated for 1 h at 47 °C for on-column trypsinization. Following incubation, peptides were eluted in three stages, all eluates pooled and peptides concentrated in a Speed-Vac (Concentrator 5301, Eppendorf, Hamburg, Germany) at 37 °C to near-dryness (~5 μL remaining).

#### 4.2.3. SDC

Ground, frozen kidney samples were processed as described in Supplementary Materials (protocols provided in Supplementary Materials Tables S1 and S2) [28]. Homogenized samples (150 µg protein) were diluted to a final volume of 62.5 μL with Denaturing Solution 1 (1.67% SDC (Sigma-Aldrich), 333.3 mM ammonium hydrogen carbonate (Fisher Scientific), and PhosSTOP phosphatase inhibitor and cOmplete Protease Inhibitor Cocktail (Roche, Basel, Switzerland) at the manufacturer’s recommended concentrations). Plasma (2.5 μL) was mixed with Denaturing Solution 1 (60 μL). Kidney and plasma samples were further denatured using 82 μL of Denaturing Solution 2 (37.6% ACN (Fisher Scientific), 2.4 mM CaCl_2_ (Fisher Scientific)) and were briefly vortexed and incubated for 5 min at room temperature before and after addition of Denaturing Solution 2. Simultaneous alkylation and reduction were performed using TCEP and CAA, as described above. Samples were digested using trypsin at a protein:trypsin ratio of 25:1, with half the trypsin added initially and samples incubated for 3 h at 42 °C, before the addition of the remaining trypsin and incubation at 37 °C for a further 16 h. Digestion was terminated and SDC precipitated simultaneously by adding formic acid (Fisher Scientific) to a final concentration of 0.2%. A final spin was performed, and the resulting supernatant was concentrated in a Speed-Vac (Concentrator 5301, Eppendorf) at 37 °C to near-dryness.

#### 4.2.4. Desalting Peptides

Peptide samples from all three methods were further purified using reverse-phase Vydac C18 Silica 96-well MACROSpin plates (SNS SS18V-L, The Nest Group Inc., Ipswich, MA, USA) as described in Supplementary Materials. Desalted peptides were concentrated in a Speed-Vac at 37 °C to near-dryness.

### 4.3. Data-Dependent and SWATH Mass Spectrometry

Retention time calibration peptides (iRT-Kit, Biognosys, Schlieren, Switzerland, Supplementary Materials) were spiked into each sample at 75× dilution of the iRT stock solution. Sequential Window Acquisition of All Theoretical Mass Spectra (SWATH-MS) was performed on a 5600+ TripleTOF mass spectrometer coupled to an ekspert nanoLC 415 system (eksigent, AB Sciex, Dublin, CA, USA) equipped with an emitter tip column packed in-house with 2.6 µm Aeris C18 material (Phenomenex, Torrance, CA, USA) on a length of 20 cm. Samples were run in both modes, Data Dependent Acquisition (DDA) and SWATH. The spectral library, as well as qualitative metrics, were obtained from DDA while we ran SWATH for the assessment of quantitative performance of the three methods. For both acquisition modes, peptides were separated over a 120 min gradient using a binary solvent system (solvent A: 1% ACN, 0.1 % FA in water, solvent B: 90% ACN, 0.1% FA in water) at a flow rate of 300 nL/min. The following linear gradient steps of solvent B were used across all samples: loading at 5% B, 3 min at 5% B, 90 min increase to 25% B, 10 min increase to 40% B, 10 min increase to 95% B, 1 min at 95% B, 1 min decrease to 5% B, 5 min at 5% B. For each sample, an MS1 survey scan was run (400 ms ion accumulation time) followed by 22 MS2 scans in which the most abundant peptides were fragmented, covering a precursor mass range of 400–1300 *m*/*z*. All high-sensitivity fragment ion scans used an accumulation time of 140 ms, resulting in a total cycle time of ~3.5 s for all 23 scans. SWATH-MS spectra were acquired using the same gradient using variable window width for precursor ion selection (Supplementary Materials Table S3). First, an MS survey scan was acquired at 100 ms accumulation time, followed by a total of 34 SWATH-MS fragment ion scans with overlapping mass/charge (*m*/*z*) windows (1 *m*/*z* for the window overlap), covering a precursor mass range of 400–1250 m/z. SWATH-MS fragment ion spectra were collected from 100–1600 *m*/*z*. The collision energy spread (CE) was automatically optimized for each window using a CE spread of 15 eV. All high-sensitivity fragment ion scans used an accumulation time of 96 ms, resulting in a total cycle time of ~3.4 s for all 35 scans. Each sample was injected three times, and the median of ion level data calculated across the three SWATH-MS runs.

### 4.4. Data Analysis

#### 4.4.1. DDA Data Quality Assessment Using SearchGUI

Raw AB Sciex *.wiff files were converted to *.mgf files using the AB Sciex MS Data Converter (Beat1.3, AB ScieX, Dublin, CA, USA) for compatibility with SearchGUI 4.1 software [30]. DDA searches were conducted using SearchGUI version 3.3.20, and peak lists obtained from MS/MS spectra identified using the X!Tandem Vengeance search engine (2015.12.15.2) [31]. Protein identification was conducted against a concatenated target/decoy [32] version of the Ovis aries (27,638 entries, 99.8%), Ovis aries musimon (46 entries, 0.2%), and Ovis aries platyura (1 entry, < 0.1%) sheep proteomes from UniProtKB [33] TrEMBL (27,685 target sequences, accessed on 22 July 2020). Precursor mass tolerance was set to 50 ppm, fragment ion tolerance to 0.2 Daltons, trypsin was selected as the protein digestion enzyme with semi-specific activity, and a maximum of 2 missed cleavages was allowed. Carbamidomethylation (+57.02 Da, Unimod #4) of cysteine was included as a fixed modification, and the variable modifications included were oxidation of methionine (+15.99 Da, Unimod #35), acetylation of the protein N-terminal (+42.01 Da, Unimod #1), and carbamidomethylation of lysine (+57.02 Da, Unimod #4). Variable modifications included during the refinement procedure were pyrrolidone from glutamic acid (−17.03 Da, Unimod #27), pyrrolidone from glutamine (+X Da, Unimod #28), and pyrrolidone from carbamidomethylated cysteine (−17.03 Da, Unimod #385). PeptideShaker version 1.16.45 [34] was used to infer peptides and proteins from the spectrum identification results. Peptide spectrum matches, peptides, and proteins were validated at a 1.0% FDR using the decoy hit distribution. Post-translational modification localizations were scored using the phosphoRS score [35] and the D-score [36] with a threshold of 95.0, as implemented in the compomics-utilities package [37]. Missed cleavage data were generated using PeptideShaker [34].

#### 4.4.2. Spectral Library Build Using ProteinPilot

DDA data for library construction were processed using ProteinPilot (v4.5, AB Sciex) due to its compatibility with the SWATH Acquisition™MicroApp pipeline used for downstream SWATH analysis. AB Sciex *wiff DDA files were searched against the above UniProt sheep proteome with the Paragon^TM^ algorithm (v4.5.0.0) and a reverse-decoy database search strategy. Search parameters were: (i) sample type: identification; (ii) cys alkylation: +57.02 Da; (iii) digestion: trypsin; iv) instrument: TripleTOF 5600; (v) special factors: none; (vi) ID focus: biological modifications; amino acid substitutions; vii) search effort: thorough ID; and (viii) detected protein threshold: > 0.05 (10.0%). This process was run three times—once for all kidney tissue DDA files, once for all plasma DDA files, and once for a single DDA file of a pooled depleted plasma sample (Supplementary Materials), resulting in the generation of three spectral libraries (comprehensive kidney tissue library, comprehensive plasma library and depleted plasma library, respectively). At an FDR of 1.0%, this yielded 1186 proteins in the tissue library, 203 proteins in the plasma library, and 231 proteins in the depleted plasma library. These spectral libraries were exported as *.group files. The SWATHXtend R package [38] was used to construct a comprehensive spectral library that merged the three libraries generated above (tissue, plasma, and depleted plasma) for use as the final spectral library when analyzing SWATH-MS data.

#### 4.4.3. SWATH Data Analysis

SWATH data were analyzed using the SWATH Acquisition™MicroApp in PeakView software, v2.2 (AB Sciex). The comprehensive spectral library was used as reference library. Possible retention time shifts between the SWATH-MS runs and library spectra were corrected by aligning the retention times of the Biognosys peptides [39] in addition to the retention time from endogenous high abundance proteins that were detected consistently across samples (peptide sequences provided in Supplementary Materials Tables S4 and S5). Processing settings in the SWATH Acquisition™MicroApp were set to the following conditions: 6 peptides per protein, 6 transitions per peptide, FDR threshold of 1.0%, a 12-min window for the extracted ion Chromatogram (XIC), and a mass accuracy of 75 ppm [40]. Shared peptides were excluded. Extracted transition ion peak areas, peptide peak areas, and protein peak areas were exported in Excel format for quality processing and statistical analysis.

#### 4.4.4. Quality Control of SWATH Data

Ion level data were processed using R (version 4.1.0) [41,42]. Samples were normalized to the ratio of the mean ion intensity (area under the curve, AUC) of all samples to the AUC of the individual sample. Ions were excluded in a sample if they were below the lower detection limit (calculated for each ion as quartile 1—(1.5× interquartile range)) or the coefficient of variation (CV) for technical injection triplicates was >25%. From triplicates, the median intensity of each ion was taken and ions derived from common precursors summed to the corresponding peptide area, and then peptides summed to protein areas [43,44,45]. Peptide and protein lists for each sample prepared using a given method were merged, resulting in one peptide- and one protein-list per method for kidney tissue, and one peptide- and one protein-list per method for plasma. For a peptide or protein to be considered reproducibly quantified in a given sample preparation method, the peptide or protein had to be quantified in all samples of the given sample type (kidney tissue or plasma) prepared using that method.

### 4.5. Statistical Analysis

All statistical analyses were performed in R (version 4.1.0) [41,42]. The number of peptides, proteins, and sequence coverage were compared between methods using ANOVA. The R Package “Peptides” (version 2.4.4) [46] was used to calculate isoelectric point (p*I*), grand average of hydropathicity (GRAVY) score, peptide length, peptide molecular weight and peptide net charge. The quantitative reproducibility of each method was evaluated by calculating Spearman correlations for the protein intensities between biological replicates and R-squared values have been reported. Protein intensities for each method were Log_2_ transformed and compared with the protein intensities from each of the other methods using t-tests [41,42]. Resulting *p*-values were corrected for multiple testing using the Benjamini-Hochberg method, and log-2 fold-changes were calculated for each protein. Principal component analysis was performed with the R Package “ggfortify” (version 0.4.14) [47]. Enrichment analysis was performed using the core and comparative workflow in Ingenuity Pathway Analysis (IPA; Qiagen Inc., https://www.qiagenbioinformatics.com/products/ingenuity-pathway-analysis, accessed on 11 January 2022). Each data set was filtered for enriched canonical pathways with a Benjamini-Hochberg-corrected *p*-value < 0.001 [24,25].

## Data Availability

The mass spectrometry proteomics data have been deposited to ProteomeXchange Consortium via the PRIDE partner repository with the dataset identifiers PXD030797 (DDA data) and PXD030796 (DIA data).

## Supplementary Materials

The following supporting information can be downloaded at: https://www.mdpi.com/article/10.3390/ijms24076290/s1, Figure S1: Protein rank graph; Figure S2: Missed cleavages; Figure S3: Median protein coverage; Figure S4: Physical characteristics; Figure S5: Median protein coverage for proteins quantified; Figure S6: Violin plots displaying the distribution of kidney tissue and plasma peptides; Table S1: Protocol of tissue trypsinization using the SDC method; Table S2: Protocol of plasma trypsinization using the SDC method; Table S3: Variable window widths for precursor ion selection; Table S4: Peptides used for retention time alignment in plasma; Table S5: Peptides used for retention time alignment in kidney tissue; Table S6: ANOVA *p*-values for physical characteristics of peptides; Table S7: ANOVA pairwise comparisons for molecular weight; Table S8: Mean molecular weight of peptides; Table S9: ANOVA pairwise comparisons for peptide length; Table S10: Mean peptide length; Table S11: Mean GRAVY scores.
