# Supplementary material for: Comparison of SPEED, S-Trap, and In-Solution-Based Sample Preparation Methods for Mass Spectrometry in Kidney Tissue and Plasma
Source: Int J Mol Sci. 2023 Mar 27;24(7):6290. doi: 10.3390/ijms24076290 (PMC10094439; doi:10.3390/ijms24076290)
Supplement: Supplementary file 1 [file ijms-24-06290-s001.zip › ijms-2223626-Supplementary.pdf]

Supplementary methods

1. SPEED protein concentrations

Corresponding protein concentration for each MacFarland standard were previously determined by Doellinger *et al.*[1] using tryptophan fluorescence measurements as a reference, allowing the calibration of any UV-spectrophotometer using commercial MacFarland standards. Measured absorbance at 360 nm (A360) was used to calculate the corresponding protein concentration in SPEED-derived samples using the equation:

$$Concentration\ of\ protein\ \left[\frac{\mu g}{\mu L}\right] = A360\ of\ sample\ x\ (\frac{0.36}{A360\ MacFarland\ 3})$$

Absorbances at 360 nm were measured for the MacFarland standard 3 (bioMérieux, OT-70900, Marcy-l'Etoile) and each sample in triplicate on a MultiSkan Go plate reader (ThermoFisher Scientific, Waltham) using SkanIt Re 5.0 software (ThermoFisher Scientific).

2. S-Trap tissue homogenisation

Ground, frozen kidney tissue (1 g) was added to a pre-chilled 7 mL Precellys tissue homogenization tube (Hard Tissue Homogenizing CK28 2 mL, P000911-LYSK0-A, Bertin Instruments, Rockville, MD) containing 2.8 mm ceramic (zirconium oxide) beads. Lysis buffer (5 mL) was added containing 0.1% sodium dodecyl sulphate (SDS, Sigma Aldrich, St. Louis), 50 mM Triethylammonium bicarbonate (Sigma-Aldrich), and PhosSTOP phosphatase inhibitor (Roche, Basel) and cOmplete Protease Inhibitor Cocktail (Roche) according to manufacturer's

recommendations (one PhosSTOP tablet per 10 mL of extraction solution, one cOmplete tablet per 50 mL extraction solution). SDS was added at 0.1% to avoid over-foaming during the homogenisation step. Samples were homogenised on a Cryolys Evolution (Bertin Instruments, Rockville, MD) at the following conditions: 4 x 15 second cycles, 30-second break between cycles, 8000 rpm, 4°C. Subsequently, samples were sonicated on ice at 30% power, 50% pulse rate for 100 seconds to shear DNA. The SDS concentration was increased from 0.1% to 5%. Samples were clarified by centrifugation at 15000 rpm for 10 minutes. Protein concentration was quantified using a Pierce bicinchoninic acid (BCA) assay (catalog #23225, ThermoFisher Scientific) following manufacturer's instructions.

3. S-Trap plasma sonication

A minimum volume of 250 µL (sample in lysis buffer) was required for adequate sonication with the available microprobe (Omni International, GA, USA). Plasma (12.5 µL containing approximately 1,875 µg of protein) was diluted to a volume of 125 µL with lysis buffer (5% SDS, 50 mM triethylammonium bicarbonate) and then further diluted to 250 µL with 2x lysis buffer containing PhosSTOP phosphatase inhibitor (Roche) and cOmplete Protease Inhibitor Cocktail (Roche) according to manufacturer's recommendations. Samples were probe sonicated on ice at 30% power, 50% pulse rate for 100 seconds. Of the 250 µL sonicated sample, a 50 µL aliquot containing 150 µg of protein was carried through the S-Trap protocol.

4. SDC tissue homogenisation

Ground tissue (1 g) was added to a pre-chilled 7 mL Precellys tissue homogenisation tube (Hard Tissue Homogenizing CK28 7 mL, P000911-LYSK0-A, Bertin Instruments, Rockville, MD) containing 2.8 mm ceramic (zirconium oxide) beads. Tissue Denaturing and Homogenising

Solution (composition listed in Supplementary Supplementary Methods **Table *S1***) was added (5 mL) containing 0.1% sodium deoxycholate (SDC), 333.3 mM ammonium hydrogen carbonate, PhosSTOP phosphatase inhibitor (Roche) and cOmplete Protease Inihibitor Cocktail (Roche) at the manufacturer's recommended concentrations (one PhosSTOP tablet per 10 mL of extraction solution, one cOmplete tablet per 50 mL extraction solution). SDC was added at 0.1% to avoid over-foaming during the homogenisation step. Samples were homogenised on a Cryolys Evolution machine (Bertin Instruments, Rockville, MD) at the following conditions: 4 x 15 second cycles, 30-second break between cycles, 8000 rpm, 4°C. Following homogenisation, the SDC concentration was increased from 0.1% to 1.67%, consistent with the method developed by Lassé *et al[2].* Samples were clarified by centrifugation at 15000 rpm for 10 minutes. Protein concentration was measured using the Pierce BCA assay following the manufacturer's instructions.

Supplementary Methods Table S1: Protocol of tissue trypsinization using the SDC method.

Solution	Composition	µL
Homogenized sheep kidney in Homogenizing and Denaturing Solution with SDC concentration topped up to 1.67%	150 µg protein	
	10% SDC	8.85
	400 mM Ammonium hydrogen carbonate	44.27
	10x stock solution PhosSTOP phosphatase inhibitor*	2.68
	25x stock solution cOmplete protease inhibitor cocktail*	6.70
Denaturing Solution 2	100% ACN	30.8
	100 mM $CaCl_2$	2.0

Solution	Composition	µL
	dH20	49.2
dH20		48
Trypsin (1)	0.5 µg/µL	6
Trypsin (2)	0.5 µg/µL	6
Formic acid	10% in dH20	40

*made in 1.67% SDC, 333.3mM ammonium hydrogen carbonate

Supplementary Methods Table S2: Protocol of plasma trypsinization using the SDC method.

Solution	Composition	µL
Sheep plasma	60 µg/µL protein	2.5
Denaturing Solution 1	10% SDC	8.55
	400 mM Ammonium hydrogen carbonate	42.77
	10x stock solution PhosSTOP phosphatase inhibitor*	2.48
	25x stock solution cOmplete protease inhibitor cocktail*	6.2
Denaturing Solution 2	100% ACN	30.8
	$CaCl_2$	2.0
	dH20	49.2
dH20		48
Trypsin (1)	0.5 µg/µL	6
Trypsin (2)	0.5 µg/µL	6
Formic acid	10% in dH20	40

* made in 1.67% SDC, 333.3mM ammonium hydrogen carbonate

5. Desalting peptides

Peptides prepared using SPEED were purified in the final solution from trypsinization containing 2% TFA, whereas dried peptides prepared using SDC or S-Trap methods were resuspended in 120 μL of 2% ACN containing 0.2% formic acid. Peptides were loaded onto the C18 resin (SNS SS18V-L, The Nest Group Inc., Ipswich) and washed three times using 200 μL 0.2% TFA (SPEED derived samples) or 2% ACN containing 0.2% formic acid (in-solution and S-Trap derived samples) with a stepped-centrifugation protocol (50 g, 110 g, 200 g and 400 g for 1 minute each, followed by 750 g for 2 minutes). Peptides were eluted using a high-organic mobile phase (0.2% formic acid in 75% ACN) using the same centrifugation protocol (50 g, 110 g, 200 g and 400 g for 1 minute each, followed by 750 g for 2 minutes).

6. Pooled, depleted plasma sample

To create a pooled, depleted plasma library, plasma samples from the three sheep were combined in equal volumes to yield 1 mL of pooled plasma and depleted using the ProteoMiner Protein Enrichment Large-Capacity Kit (#263-3007, BioRad, Hercules) following the manufacturer's instructions. Briefly, ProteoMiner columns were washed twice with 600 μL of wash buffer before loading with 1 mL of plasma sample. Loaded columns were rotated and incubated at room temperature for 1 hour before washing three times with 600 μL of wash buffer. Samples were eluted in 200 μL of elution agent.

7. SWATH mass spectrometry

Table S3. Variable window widths for precursor ion selection in SWATH mass spectrometry

Window	Start mass (Da)	Stop mass (Da)
1	399.5	458.5
2	457.7	496.8
3	495.8	521.5

4	520.5	538.7
5	537.7	554.4
6	553.4	570.2
7	569.2	586.8
8	585.8	606.2
9	605.2	625.1
10	624.1	642.2
11	641.2	656.6
12	655.6	669.6
13	668.6	683.1
14	682.1	696.2
15	695.2	710.1
16	709.1	726.3
17	725.3	745.2
18	744.2	764.1
19	763.1	783.9
20	782.9	802.4
21	801.4	820.8
22	819.8	839.7
23	838.7	859.1
24	858.1	880.7
25	879.7	904.1
26	903.1	927.5
27	926.5	952.2
28	951.2	977.4
29	976.4	1004.0
30	1003.0	1035.9
31	1034.9	1076.0
32	1075.0	1122.8
33	1121.8	1176.3
34	1175.3	1249.7

8. Retention time alignment

Table S4. Peptides used for retention time alignment in plasma

Protein	Peptide	Expected RT
Biognosys iRT peptides	LFLQFGAQGSPFLK	97.46
	ADVTPADFSEWSK	68.92
	TPVISGGPYEYR	48.94
	GAGSSEPVTGLDAK	26.62
	GTFIIDPGGVIR	77.82
	TPVITGAPYEYR	52.52

	GTFIIDPAAVIR	87.59
Plasminogen W5P3R3	EQQC[CAM]VIMGGSSK	20.82
	GHIFTPETNPR	21.20

Table S5. Peptides used for retention time alignment in kidney tissue

Protein	Peptide	Expected RT
LDL receptor related protein 2 OS=Ovis aries OX=9940 GN=LRP2 PE=4 SV=1	TVLVSDITATPR	51.37
	TIIQNLNNPR	34.87
	ISIEGGESEVIR	49.24
	TGSILPSLPK	56.64
	IYFTQLLPSGK	81.93
	LLTVNPWLTQVR	85.49
	YLVQPPGLAVDWVGR	91.05
Serum albumin OS=Ovis aries OX=9940 GN=ALB PE=4 SV=1	RHPEYAVSVLLR	42.68
	ADFTDVTK	24.50

Supplementary Figures

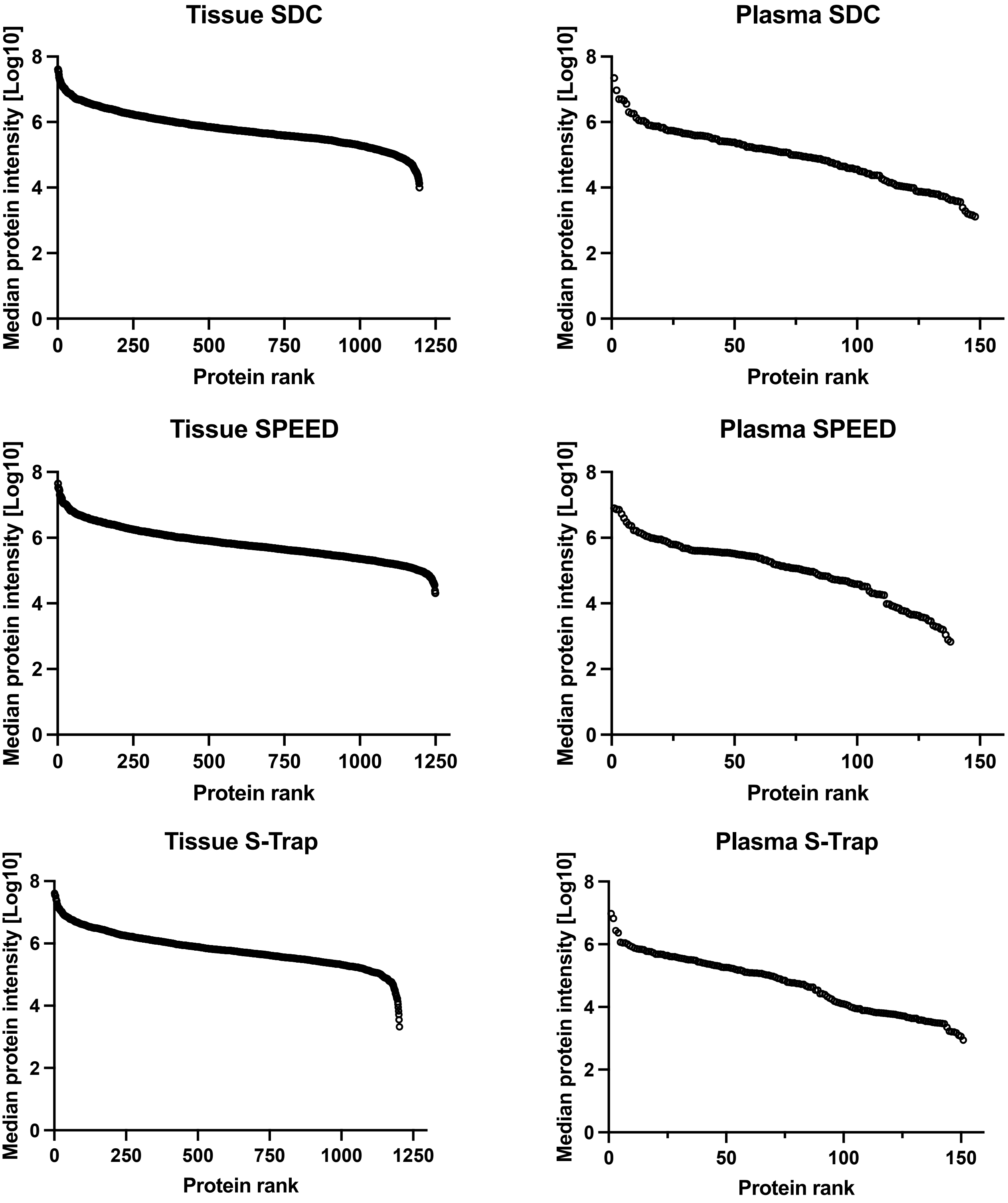

Supplemental Figure S1. Median protein intensity for proteins quantified using each method, ranked from most abundant to least abundant. In tissue, S-Trap quantified proteins across five orders of mass spectrometry signal magnitude, and SDC and SPEED quantified proteins across four orders. All three methods quantified proteins across five orders of magnitude in plasma.

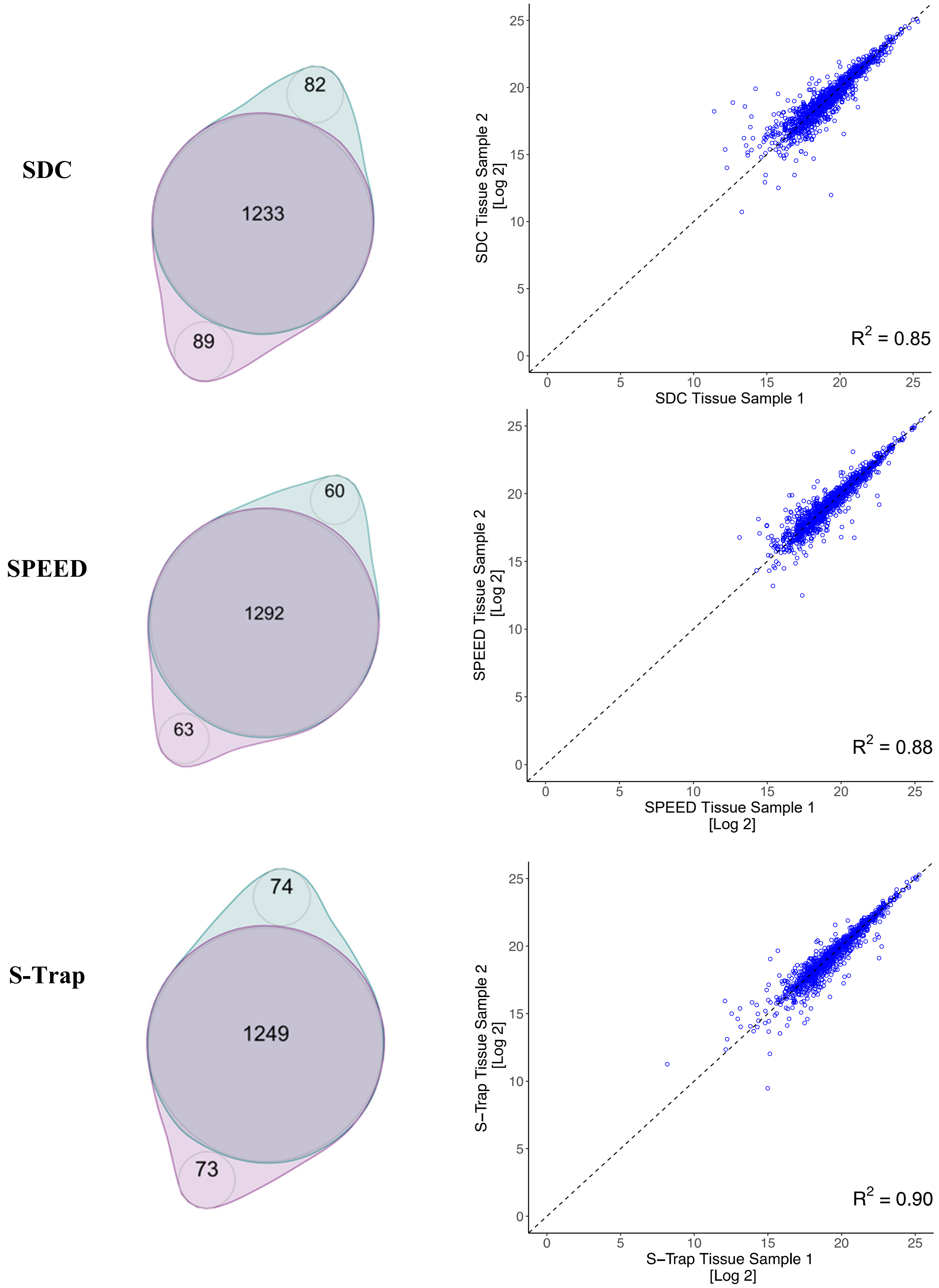
SDC
82
1233
89
SPEED
60
1292
63
S-Trap
74
1249
73
SDC Tissue Sample 2 [Log 2]
SDC Tissue Sample 1
R2 = 0.85
SPEED Tissue Sample 2 [Log 2]
SPEED Tissue Sample 1 [Log 2]
R2 = 0.88
S−Trap Tissue Sample 2 [Log 2]
S−Trap Tissue Sample 1 [Log 2]
R2 = 0.90
0
5
10
15
20
25

Supplemental Figure S2. Reproducibility of proteins quantified using SWATH in biological replicates in kidney tissue. Venn diagrams display the overlap in the identities of proteins quantified in two biological replicates for each preparation method. Scatter plots display the relative protein intensities of proteins quantified in both biological replicates and the corresponding Pearson R-squared value for the correlation. Dashed black lines represent the line of perfect correlation, R^2=1.

SDC

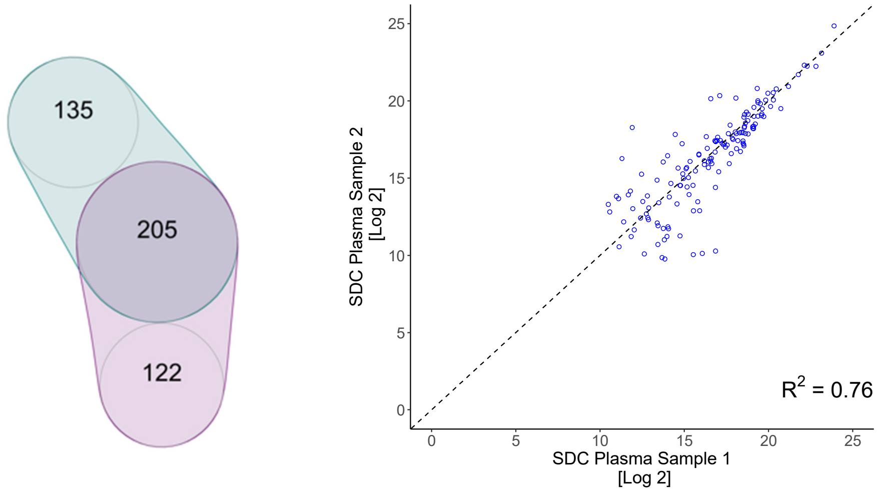

SPEED

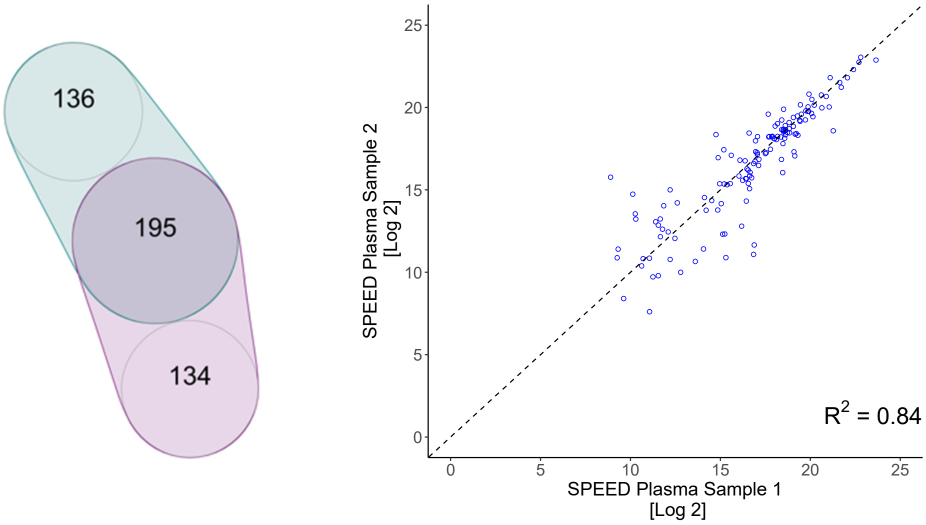

S-Trap

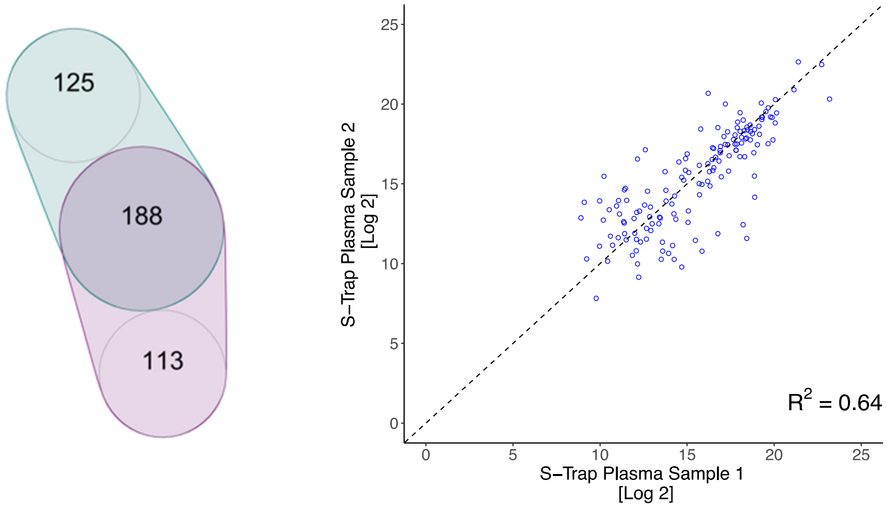

Supplemental Figure S3. Reproducibility of proteins quantified between biological replicates in plasma. Venn diagrams display the overlap in the identities of the proteins quantified across two biological replicates for each preparation method. Scatter plots display the relative protein intensities of proteins quantified in both biological replicates and the corresponding Pearson R-squared value for the correlation. Dashed black lines represent the line of perfect correlation, $R^2=1$.

A)

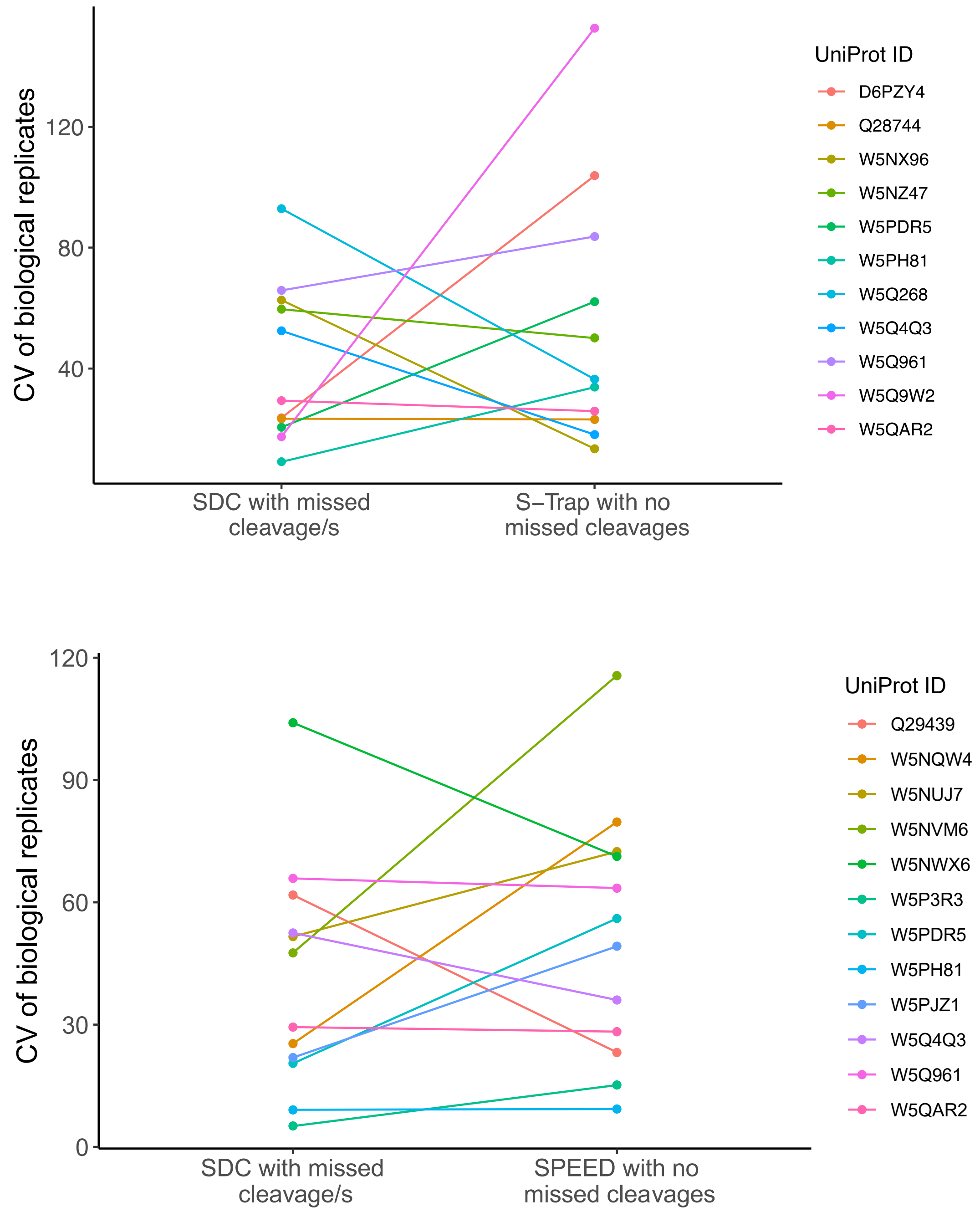

Supplemental Figure S4. Plots indicating that the greater number of missed cleavages observed for the in-solution method does not increase the variability of protein quantifications, compared with S-Trap or SPEED. The coefficient of variation (CV) for protein quantifications across

biological replicates was compared between each method. One-way ANOVA were performed to compare the CV of proteins prepared using in-solution with missed cleavages versus the same proteins prepared using A) SPEED or B) S-trap with no missed cleavages. This revealed no significant differences in the CVs of protein quantifications between methods (in-solution with missed cleavages versus S-Trap with no missed cleavages p=0.39; in-solution with missed cleavages versus SPEED with no missed cleavages p=0.40). Moreover, none of the proteins prepared using in-solution with missed cleavages or using S-Trap or SPEED with no missed cleavages were differentially quantified between the methods (**article Figure 3)**. This provides further evidence that missed cleavages do not affect the quantification of proteins in SWATH-MS.

A)

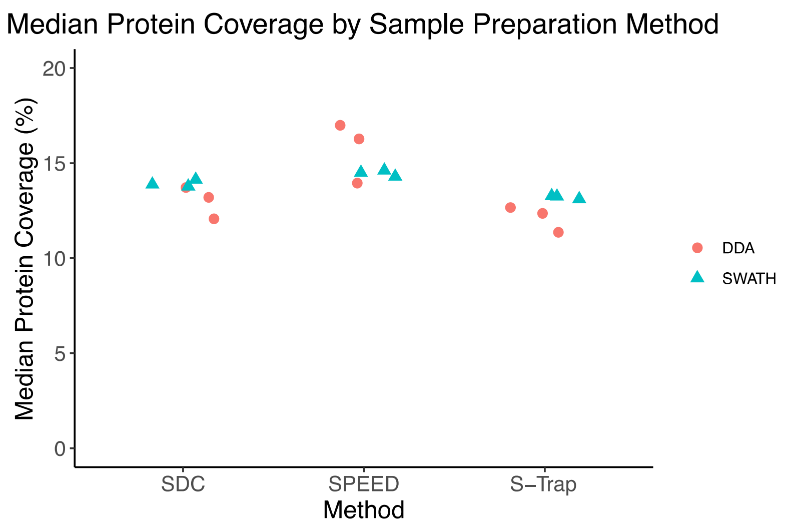

B)

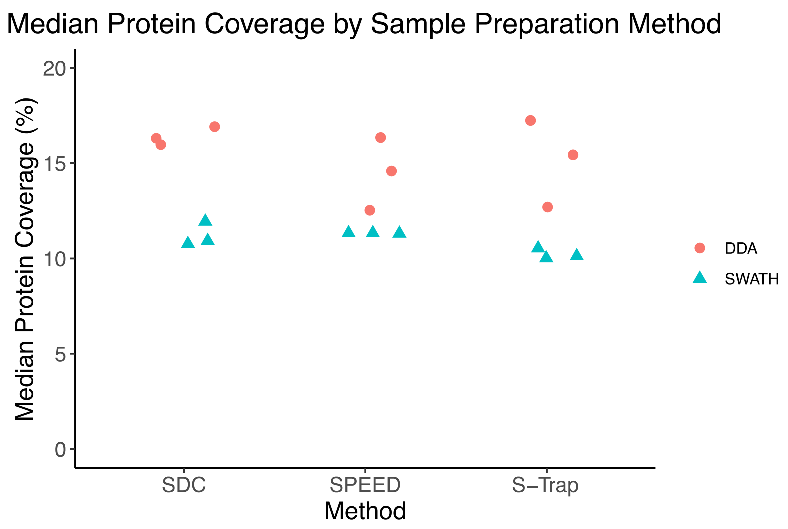

Supplemental Figure S5. Median protein coverage for proteins quantified using SWATH-MS and identified in DDA for in-solution, SPEED and S-Trap methods in A) kidney tissue and B) plasma. Each point represents a single sample and the median coverage of proteins quantified (SWATH-MS, indicated by red circles) or detected (DDA, indicated by teal triangles) within it.

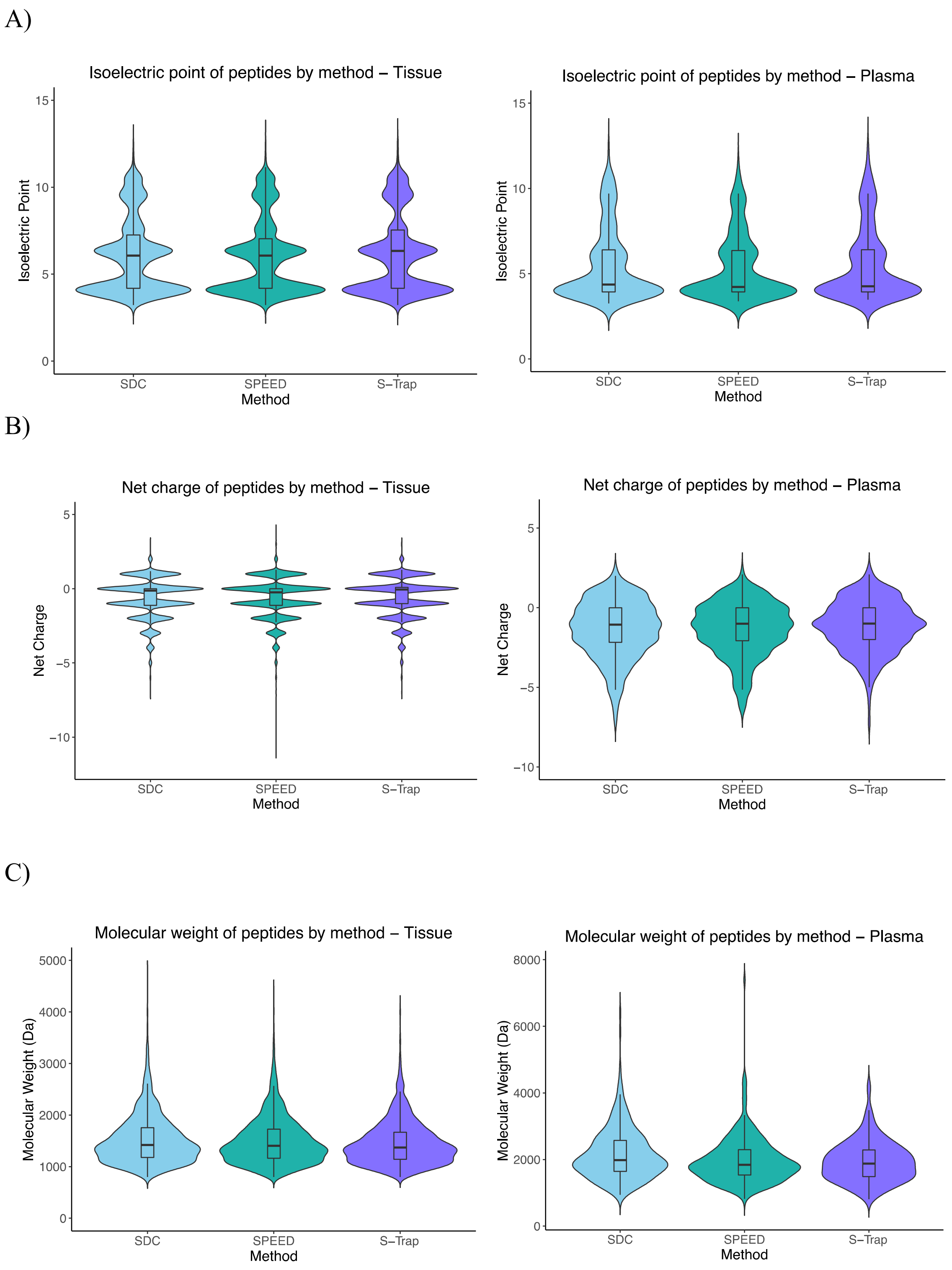
A)
Isoelectric point of peptides by method – Tissue
Isoelectric Point
Method
SDC
SPEED
S–Trap
Isoelectric point of peptides by method – Plasma
B)
Net charge of peptides by method – Tissue
Net Charge
Net charge of peptides by method – Plasma
C)
Molecular weight of peptides by method – Tissue
Molecular Weight (Da)
Molecular weight of peptides by method – Plasma

D)

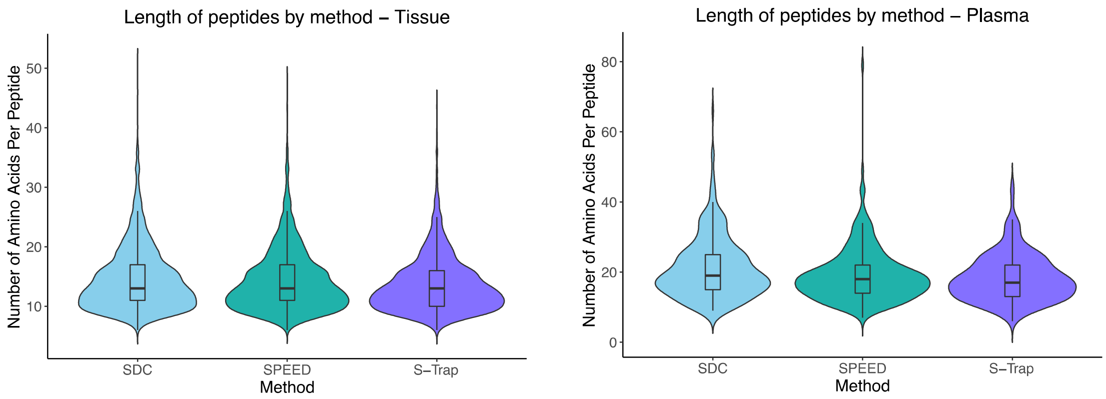

E)

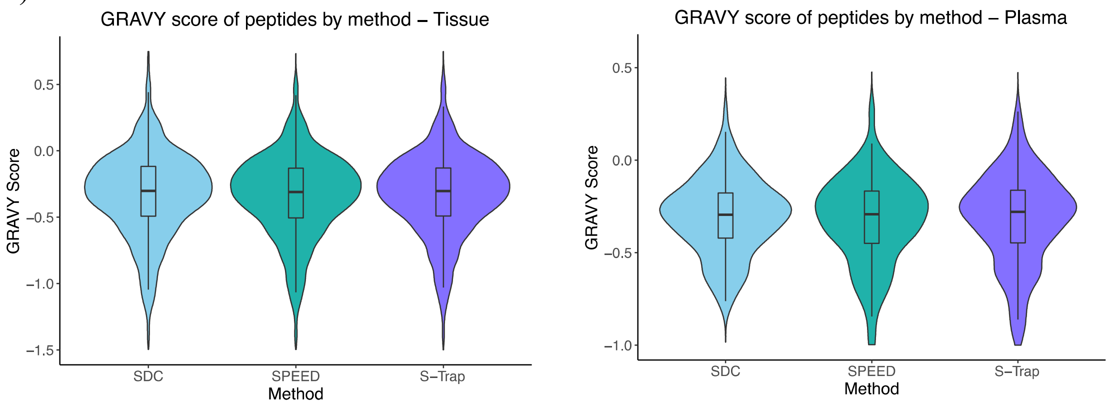

Supplemental Figure S6. Violin plots displaying the distribution of kidney tissue and plasma peptides for A) isoelectric point, B) net charge, C) molecular weight, D) length and D) GRAVY score. Medians and interquartile ranges shown.

Supplemental Table S6. ANOVA p-values for physical characteristics of peptides detected by each method, in both plasma and tissue.

Physical characteristic	Plasma p-value	Tissue p-value
Isoelectric point	0.450	0.083
Net charge	0.238	0.238
Molecular weight	<0.001	<0.001
Peptide length	<0.001	<0.001
GRAVY score	0.994	0.829

Supplemental Table S7. ANOVA pairwise comparisons for molecular weight between methods presented for plasma and tissue

Methods	Plasma p-value	Tissue p-value
In-solution v S-Trap	<0.001	<0.001
In-solution v SPEED	0.002	0.012
SPEED v S-Trap	0.485	<0.001

Supplemental Table S8. Mean molecular weight (Daltons) by method, for plasma and tissue

Method	Plasma mean (±SD) molecular weight (Da)	Tissue mean (±SD) molecular weight (Da)
In-solution	2167 (±778)	1524 (±483)
SPEED	1981 (±705)	1499 (±464)
S-Trap	1938 (±608)	1456 (±431)

Supplemental Table S9. ANOVA pairwise comparisons for peptide length between methods presented for plasma and tissue

Methods	Plasma p-value	Tissue p-value
In-solution v S-Trap	<0.001	<0.001
In-solution v SPEED	0.004	0.009
SPEED v S-Trap	0.205	<0.001

Supplemental Table S10. Mean peptide length (number of amino acids) by method for plasma and tissue

Method	Plasma mean (±SD) peptide length	Tissue mean (±SD) peptide length
In-solution	21.1 (±8.7)	14.6 (±5.3)
SPEED	19.2 (±7.8)	14.3 (±5.2)
S-Trap	18.3 (±6.7)	13.6 (±4.6)

Supplemental Table S11. Mean GRAVY scores for samples prepared using each method in plasma and tissue

Method	Plasma mean (±SD) GRAVY score	Tissue mean (±SD) GRAVY score
In-solution	-0.325 (±0.25)	-0.334 (±0.33)
SPEED	-0.326 (±0.23)	-0.341 (±0.32)
S-Trap	-0.328 (±0.26)	-0.334 (±0.32)

1. Doellinger, J.; Schneider, A.; Hoeller, M.; Lasch, P., Sample Preparation by Easy Extraction and Digestion (SPEED) - A Universal, Rapid, and Detergent-free Protocol for Proteomics Based on Acid Extraction. *Mol Cell Proteomics* **2020,** 19, (1), 209-222.
2. Lasse, M.; Pilbrow, A. P.; Kleffmann, T.; Andersson Overstrom, E.; von Zychlinski, A.; Frampton, C. M. A.; Poppe, K. K.; Troughton, R. W.; Lewis, L. K.; Prickett, T. C. R.; Pemberton, C. J.; Richards, A. M.; Cameron, V. A., Fibrinogen and hemoglobin predict near future cardiovascular events in asymptomatic individuals. *Sci Rep* **2021,** 11, (1), 4605.
